# Low risk of acquiring melioidosis from the environment in the continental United States

**DOI:** 10.1371/journal.pone.0270997

**Published:** 2022-07-29

**Authors:** Carina M. Hall, Daniel Romero-Alvarez, Madison Martz, Ella Santana-Propper, Lora Versluis, Laura Jiménez, Abdelghafar Alkishe, Joseph D. Busch, Trevor Maness, Jonathan Stewart, Tom Sidwa, Jay E. Gee, Mindy G. Elrod, Zachary Weiner, Alex R. Hoffmaster, Jason W. Sahl, Johanna S. Salzer, A. Townsend Peterson, Amanda Kieffer, David M. Wagner

**Affiliations:** 1 Pathogen Microbiome Institute, Northern Arizona University, Flagstaff, Arizona, United States of America; 2 University of Kansas, Lawrence, Kansas, United States of America; 3 OneHealth Research Group, Facultad de Medicina, Universidad de las Américas, Quito, Ecuador; 4 Texas Department of State Health Services, San Antonio, Texas, United States of America; 5 Texas Department of State Health Services, Austin, Texas, United States of America; 6 Centers for Disease Control and Prevention, Atlanta, Georgia, United States of America; University of Toledo College of Medicine and Life Sciences, UNITED STATES

## Abstract

Melioidosis is an underreported human disease of tropical and sub-tropical regions caused by the saprophyte *Burkholderia pseudomallei*. Although most global melioidosis cases are reported from tropical regions in Southeast Asia and northern Australia, there are multiple occurrences from sub-tropical regions, including the United States (U.S.). Most melioidosis cases reported from the continental U.S. are the result of acquiring the disease during travel to endemic regions or from contaminated imported materials. Only two human melioidosis cases from the continental U.S. have likely acquired *B*. *pseudomallei* directly from local environments and these cases lived only ~7 km from each other in rural Texas. In this study, we assessed the risk of acquiring melioidosis from the environment within the continental U.S. by surveying for *B*. *pseudomallei* in the environment in Texas where these two human melioidosis cases likely acquired their infections. We sampled the environment near the homes of the two cases and at additional sampling locations in surrounding counties in Texas that were selected based on ecological niche modeling. *B*. *pseudomallei* was not detected at the residences of these two cases or in the surrounding region. These negative data are important to demonstrate that *B*. *pseudomallei* is rare in the environment in the U.S. even at locations where locally acquired human cases likely have occurred, documenting the low risk of acquiring *B*. *pseudomallei* infection from the environment in the continental U.S.

## Introduction

*Burkholderia pseudomallei* is a Gram-negative saprophytic bacterium and the causative agent of the tropical and sub-tropical human disease, melioidosis [1–3]. Infections with *B*. *pseudomallei* are usually acquired from the environment via contaminated soil or water with percutaneous inoculation and inhalation as the main routes of transmission [4]. Ingestion is a less common route but it does occur [5–7]; person-to-person transmission is extremely rare [8]. The majority of melioidosis cases are reported from Southeast Asia and northern Australia although the global disease burden is thought to be drastically underestimated [9]. There has been a recent increase in reports of locally acquired melioidosis from sub-tropical regions, such as Sonora Mexico [10], Western Australia [11], and Texas in the United States (U.S) [12–14]. In the continental U.S. (CONUS) human melioidosis cases can be assigned to three categories in order of frequency: 1) acquired outside the CONUS during travel to endemic regions (*e*.*g*., the Caribbean, Central/South America, Southeast Asia), 2) acquired within the CONUS from contaminated items imported from endemic regions, and 3) acquired directly from the local environment in the CONUS (Table 1). Rare laboratory-acquired infections can also occur [15, 16].

**Table 1:** Published melioidosis cases reported from the CONUS since 1948 (excluding laboratory acquired infections).

Type of case*	Year	State	Source of infection	Phylogenetic origin of source	Outcome	Citation
1	1948–2015 (*n* = 117)	various	various around the world	various around the world	various	[15, 17–19]
2	1950	unknown	unknown	unknown	survived	[20]
2	1971	California	potential in utero	suspected SE Asia	died	[3]
2	1973	Mississippi	sexually transmitted	suspected SE Asia	survived	[2]
2 or 3	1999	Arizona	potential travel to El Salvador	Americas	survived	[12]
**3**	**2004**	**Texas**	**suspected local environment**	**Americas**	survived	[13, 14]
2	2008	Arizona	unknown	SE Asia	survived	[21]
2	2010	California	suspected reptile	unknown	survived	[15]
2	2013	Ohio	unknown	SE Asia	died	[22]
**3**	**2018**	**Texas**	**suspected local environment**	**Americas**	survived	[12]
2	2019	Maryland	fish from a home aquarium	SE Asia	survived	[23]
1	2020	Arizona	potential travel to Dominican Republic	Caribbean	survived	[24]
2	2021	Kansas	suspected aromatherapy room spray	SE Asia	died	[25]
2	2021	Texas	suspected aromatherapy room spray	SE Asia	survived	[25]
2	2021	Minnesota	suspected aromatherapy room spray	SE Asia	survived	[25]
2	2021	Georgia	aromatherapy room spray	SE Asia	died	[25]

*The type of melioidosis case can be an infection acquired 1) outside the CONUS while traveling to an endemic region, 2) inside the CONUS from a contaminated source imported from outside the contiguous U.S., or 3) inside the CONUS from the local environment (bolded text).

Melioidosis was documented in many U.S. military personnel returning from the Vietnam War [26], and travel to endemic regions remains the most common source of melioidosis in residents of the CONUS [15]. Multiple types of contaminated imported products, including potting soil, iguanas, ornamental fish, and medical supplies, have been implicated as potential or confirmed sources of human infections in residents of the CONUS with no history of travel to endemic regions [15, 23]. From March-July 2021, four human melioidosis cases, including two fatalities, occurred in Georgia, Kansas, Minnesota, and Texas; these individuals had no contact with one another. Genomic analysis of *B*. *pseudomallei* case isolates indicated they were clonal and most closely related to isolates from South Asia. The investigation implicated an imported bottle of aromatherapy spray as the *B*. *pseudomallei* source [25].

To date, only two human melioidosis cases in the CONUS have likely originated from *B*. *pseudomallei* present in local environments. Both cases were reported from Atascosa County, Texas, in 2004 and 2018 [12, 14]; the residences of these two cases are located only ~7 km apart. The first case had no travel history to endemic regions except for time in Southeast Asia as a prisoner during World War II, 62 years before his melioidosis diagnosis. It was long assumed he was exposed to *B*. *pseudomallei* during this time in Southeast Asia, leading to a latent infection of melioidosis [14]. However, a recent phylogeographic analysis of the infecting *B*. *pseudomallei* strain revealed it was most closely related to isolates from the Western Hemisphere, not isolates from Southeast Asia, suggesting he acquired melioidosis locally, and more recently, in Texas [13]. In 2018, a second man from Atascosa County was diagnosed with melioidosis; he had no significant travel history in the previous 30 years. Phylogeographic analysis of both infecting *B*. *pseudomallei* strains from Texas grouped them together, suggesting a recent common ancestor for these strains, and placed them within a larger phylogenetic clade consisting solely of isolates from the Western Hemisphere. In response to the 2018 case, 56 environmental samples were collected that same year in and around his house; all were negative for *B*. *pseudomallei* [12].

To examine the risk of human melioidosis cases originating from environments in the CONUS, we conducted additional environmental sampling near and around the residences of the two melioidosis cases from Atascosa County, Texas, which are the only locally acquired melioidosis cases from the CONUS to date. When no *B*. *pseudomallei* was detected in any of these samples, we sampled from counties surrounding Atascosa County using ecological niche modeling to select additional sampling sites based upon areas predicted to contain suitable habitat for *B*. *pseudomallei*.

## Results

All 370 environmental samples collected from Texas were negative for the presence of *B*. *pseudomallei* DNA. Other *Burkholderia* spp. were identified from these environmental samples using species-specific TaqMan assays and subsequently isolated, demonstrating that the methods used were robust. Further supporting the validity of the methods used in this study is the previous identification and isolation of *B*. *pseudomallei* from soils in Puerto Rico and the U.S. Virgin Islands using these same methods [27, 28]. Most soil samples collected in Texas in 2019 were moist, whereas the majority of soil samples collected in Texas in 2020 were extremely dry.

## Discussion

We did not detect *B*. *pseudomallei* in any of the samples collected from Texas, nor was *B*. *pseudomallei* detected in previous studies conducted in this region [12]. This does not confirm that *B*. *pseudomallei* is absent from the environment in this region of Texas but does indicate that if it is present in the environment, it is rare, as expected given the limited number of cases contracted from the environment (Table 1). A lack of travel history for the two Texas cases and phylogeographic analysis of the infecting strains strongly suggest these two infections were independently acquired from the local environment in Texas. Detecting *B*. *pseudomallei* in the environment has been historically challenging due to the incomplete understanding of its optimal habitat (*e*.*g*., soil composition, moisture content, presence of competing bacteria [29–31]). However, our findings are consistent with the idea that *B*. *pseudomallei* is quite rare in the environment in this region of Texas. This is similar to Puerto Rico and the U.S. Virgin Islands, where *B*. *pseudomallei* sporadically causes human cases but is only rarely detected in the environment [27, 28].

Although rare, it is possible that *B*. *pseudomallei* is persisting in the environment in Texas, as the two patients acquired melioidosis 14 years apart and only ~7 km from each other [12, 14]. The mechanism that allows *B*. *pseudomallei* to persist in arid regions is poorly understood, although it has been speculated that a latent state may play a role in the persistence of *B*. *pseudomallei* in these situations, based upon a study from Western Australia that demonstrated limited genetic changes in *B*. *pseudomallei* in an arid region over a >50 year period [11]. Additionally, melioidosis cases from arid regions are typically reported following extreme weather events with heavy rainfall [10, 11], suggesting water could be an activator of dormant cells, or perhaps heavy rainfall allows *B*. *pseudomallei* near the underground water table to migrate towards the surface from deeper soils. If a better understanding of how and where *B*. *pseudomallei* persists in the environment in arid regions, such as Texas, can be developed, then it could help mitigate future melioidosis cases, such as advising the treatment of well water [32] or avoiding playing or swimming in pooled water after flooding events [10].

## Conclusions

The findings from our environmental sampling efforts in Texas, together with the lack of evidence to date for other human melioidosis cases in the CONUS originating from local environmental sources, suggest the risk of acquiring melioidosis from the environment in the CONUS is currently quite low. That said, only very limited environmental sampling has been performed to search for *B*. *pseudomallei* in the environment in the CONUS. Future efforts should focus on searching for *B*. *pseudomallei* in the environment in regions where it is predicted to likely occur within the CONUS (S3 Fig), such as the southeastern U.S.

## Material and methods

### Environmental sampling

**Fig 1:**
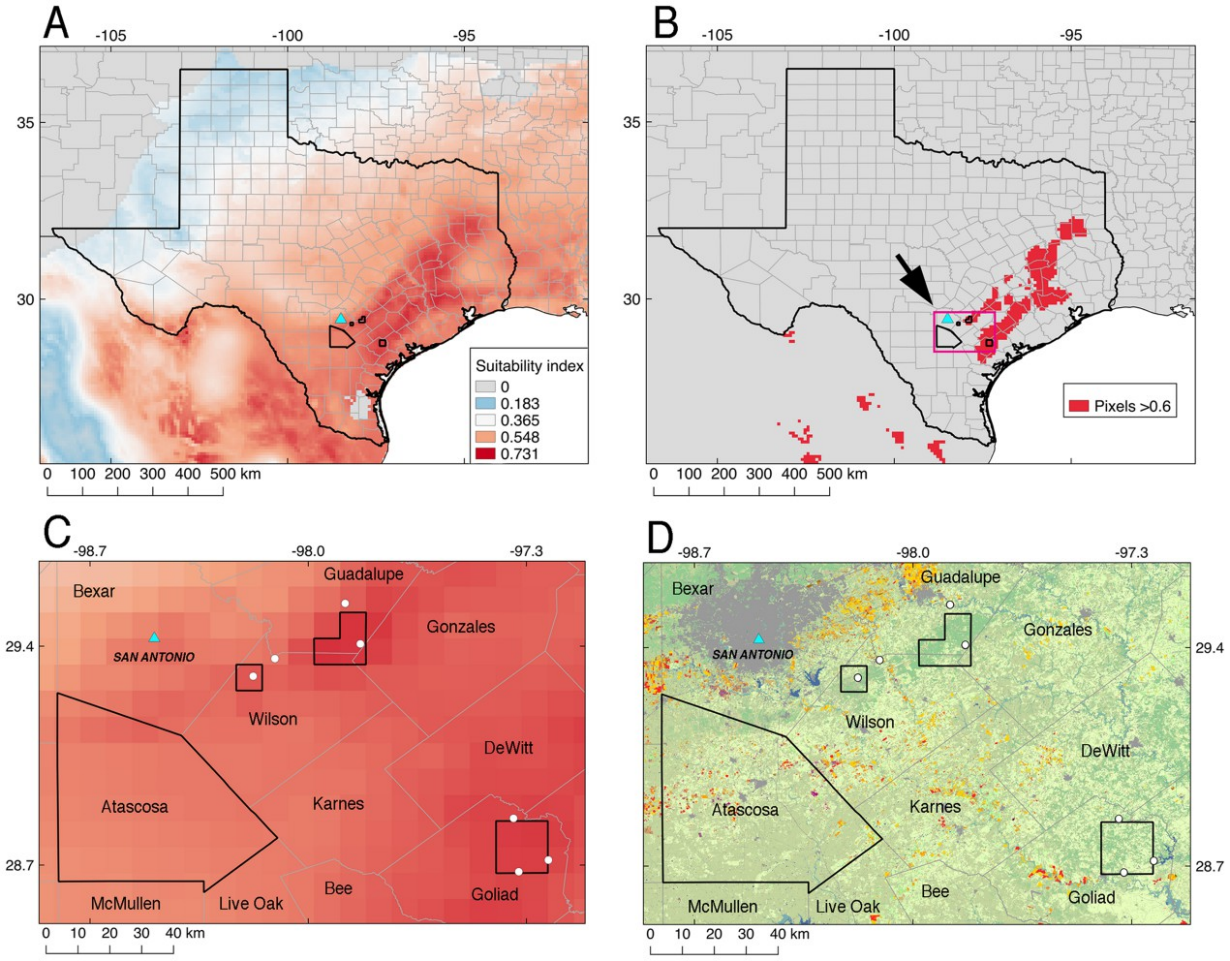
Ecological niche modeling for *B*. *pseudomallei* transferred to Texas to inform environmental sampling. For reference, the light blue triangle represents San Antonio, Texas and the polygon south of San Antonio represents Atascosa County, where two locally acquired human melioidosis cases were reported. (A) Selected continuous output of the ecological niche model. (B) Binary map showing highly suitable pixels in red, defined as those with a suitability index above 0.6. (C) Zoom-in to the inset on panel B showing the pixels (polygons other than the one representing Atascosa County) with the highest suitable values that are the most proximal to Atascosa County selected for environmental sampling (white circles = actual individual sampling sites). (D) Same as panel C using the Cropland Data Layer from the NASS CropScape geospatial portal from the USDA National Agricultural Statistics, representing the 2019 product with a resolution of 30 m (Available through: https://nrcs.app.box.com/v/gateway/folder/22218925171; [33]). Most representative land cover classes include: gray = developed, orange = sorghum plantations, red = cotton plantations, light green = mixed and deciduous forest, pale leaf = shrubland.

A total of 380 environmental samples were collected in Texas during one of two sampling events, one in November 2019 and another in November 2020. A total of 210 environmental samples were collected in Atascosa County, Texas in 2019, including 120 soil samples collected from eight sites, 80 water samples from eight sites, and 10 environmental scrapes from one site. To expand the geographic extent of our sampling, in 2020 we collected 160 additional soil samples from three counties surrounding Atascosa County (Guadalupe, Goliad, and Wilson; Fig 1). The selection of the six additional sampling sites was based upon areas predicted by ecological niche models to be suitable habitats for *B*. *pseudomallei* (Fig 1, S1 Appendix) and conditioned on available access.

Environmental sampling was conducted using methods previously described [27] and was adapted from international consensus guidelines [34] with additional modifications developed by the Menzies School of Health and Research in Darwin, Australia [35]. Permission was obtained from landowners to collect soil and/or water samples on their property when required. For each site, 10–30 soil samples were collected at a depth of 30 cm from holes spaced 2.5 m apart in one to two 10-hole transects or in a grid as previously described [27]. A broad characterization of the soil moisture content was recorded for each soil sample. Water samples (150 mL) were collected along a linear transect with 2.5 meters between each sampling location, when possible. At each site where water samples were collected, 10 samples were collected ~1 m from the water’s edge. If a site had flowing water, then water was collected near the water edge to avoid disturbance from the current. All water samples were filtered on the same collection day using a Sartorius water filtration manifold with 0.22 μm nitrocellulose filters as previously described [27]. Environmental scrapes were collected at one site from a partially empty residential 500-gallon water holding tank. Briefly, the bottom and sides of the tank were scraped, and the scrape contents were placed into a sterile 2 mL screw-cap tube. All samples were kept from direct UV exposure, stored, and shipped at ambient temperature to Northern Arizona University for further processing. Upon arrival, samples were stored in the dark at ambient temperature. The appropriate permits were obtained from the US Department of Agriculture’s Animal and Plant Health Inspection Service and the Arizona Department of Agriculture, and permit conditions were adhered to while shipping soil samples from Texas and processing them in Arizona.

### Molecular detection of *B*. *pseudomallei*

To prepare the environmental scrapes for culturing, the 2 mL tubes containing the scrapes were first vortex at high speed for 1 minute and then sonicated for 5 minutes using a Branson sonicator bath set to 70W, 42kHz at room temperature. All samples were processed in the same way for the detection and isolation of *Burkholderia* spp. as previously described [27] with the following modifications. Each water sample was filtered onto one filter, which was then cut in half and only one half was used for the inoculation of 30 mL of Ashdown’s broth. The entire contents of the environmental scrape were transferred to the 30 mL of Ashdown’s broth. The soil was processed in the same way as Hall *et al*., 2019 [27] except the soil was added straight into 30 mL of Ashdown’s broth instead of first into water. Methods for Ashdown’s broth processing, DNA extraction, and PCR screening can be found in Hall *et al*., 2019 [27]. Briefly, after 48 hours of incubating at 37°C while shaking, 1mL of the Ashdown’s broth was pelleted and DNA was extracted using QIAamp Fast DNA Stool Mini Kit (QIAGEN, Germantown, MD) following the manufacturer’s instructions, after the first re-suspending the broth pellet with 1mL of the InhibitEX Buffer. The microbial community in the Ashdown’s broth was screened for the presence of *B*. *pseudomallei* by screening undiluted DNA in triplicate using a real-time TaqMan PCR assay that targets *orf2* in the type three secretion system 1 (TTS1) cluster of *B*. *pseudomallei* [36].

## Supporting information

S1 Appendix The methods and results of the ecological niche modeling that were used for the selection of additional sampling sites with a suitable habitat for *B*. *pseudomallei*. (PDF)

S1 Fig Study area for developing the ecological niche model. To construct the accessible area used as a model calibration area (pink), we used a ratio of 500 km around 28 recent environmental soil isolations starting in the 2000s obtained from [9]. Location data are available in that reference and can be obtained following the filtering process described in the methods section.
(TIF)

S2 Fig Mobility-oriented parity metric depicting areas of strict extrapolation (dark purple) and gradients of relative similarity between the calibration (S1 Fig) and transfer area. Model interpretation in the purple areas would be highly inadvisable.
(TIF)

S3 Fig Potential distribution of *Burkholderia pseudomallei* in the 48 contiguous United States. Maps show the continuous model output (upper panel) and the uncertainty is represented as the interquartile range (IQR) among bootstrap replicates (bottom panel). Gray represents areas of strict extrapolation automatically set to zero by the algorithm.
(TIF)

S1 Table Environmental variables and percent of variance explained by each of the first three principal components (PC1-3). PCs included in the analyses are highlighted in gray.
(PDF)

S2 Table Maxent parameters for the selected best models and the values corresponding to their evaluation metrics. OR = omission rates, pROC = partial receiving operating characteristic curve (ROC), AICc = Akaike Information Criterion corrected for sample size.
(PDF)
