# Supplementary material for: Low risk of acquiring melioidosis from the environment in the continental United States
Source: PLoS One. 2022 Jul 29;17(7):e0270997. doi: 10.1371/journal.pone.0270997 (PMC9337633; doi:10.1371/journal.pone.0270997)
Supplement: S1 Appendix — (PDF) [file pone.0270997.s001.pdf]

**S1 Appendix. The methods and results of the ecological niche modeling that were used for the selection of additional sampling sites with a suitable habitat for *B. pseudomallei*.**

# Methods

## Ecological niche modeling (ENM)

Suitable areas were characterized for *B. pseudomallei* globally using ecological niche modeling and continuous model outputs were used to guide the selection of sampling sites in Texas, United States (U.S.). Selection was based on the highest values of suitability identified by the model and proximity to residences of the 2004 and 2018 melioidosis human cases identified in Texas (specific locations not shown to protect the privacy of these individuals).

### Occurrence data

Occurrence records were obtained from the supplementary material of the global summary provided by Limmathurotsakul et al. [1]. Among these records, we constrained data collection to points from the year 2000 and later, considering the timeframe of our environmental predictor datasets [2]. We used occurrences labeled as endemic (i.e., OCCURRENCE_EXPORT = No) and as isolated from soil sources (i.e., OCCURRENCE_TYPE = Bps in soil; [1]). We applied a filtering process involving elimination of duplicates, and a spatial thinning filter of 30 km to avoid over-representation of environments caused by geographic clusters of sampling [3-5]; the latter step was achieved using the spThin package in R [6]. We ended up with 28 occurrences distributed across Oceania, Southeast Asia, India, and northern Brazil (S1 Fig).

## Hypothesis of dispersal (M)

A crucial step in ecological niche modeling is the definition of a hypothesized accessible area for the studied species (M, sensu [7]), which accounts for its dispersal potential in model development. The definition of M impacts both modeling and downstream analysis, determining, among other things, the ability of the models to retrieve useful information [8, 9]. Lacking a standardized methodology to delimit M, we rely on proxies of dispersal such as distances from known occurrences [10, 11], invasive species fronts [12], or ecoregions [13, 14]. The ability of *B. pseudomallei* to survive in soils and become aerosolized allows us to assume a dispersal potential broader than the localities where it has been found [1, 15-17]. As such, we used a merged buffer of ~500 km around the points collected for this study to obtain enough environmental signal for model calibration to transfer outputs to the entire globe (pink areas in S1 Fig).

## ENM environmental variables

Three sets of environmental predictors were used, related to temperature, humidity, and soils. For temperature and humidity, data layers were used from the open-access repository of satellite-derived bioclimatic variables MERRAclim [18], eliminating the four variables that combine temperature and humidity (i.e., BIO 8, 9, 18, and 19), since they are known to hold odd spatial discontinuities that do not correspond to actual, on-ground climatic breaks [19, 20]. Nine 9 temperature-related variables and six humidity-related variables were used at a 5' resolution (~8 km spatial resolution; S1 Table; [18]).

Soil predictors were derived from the SoilGrids database (https://www.isric.org/explore/soilgrids), an open-access source with worldwide information of physical, chemical, and site characteristics of soils at 250 m resolution. We obtained four soil-

based variables, including clay content, coarse fragments, pH index, and sand content, each at two depths (0-5 cm, 15-30 cm), for a total of eight environmental predictors [21]. The soil variable data layers were resampled to match the spatial resolution of the MERRAclim variables (i.e., 5').

A principal components analysis (PCA) was applied to each set of environmental variables based on a singular value decomposition [22] using the '*kuenm_rpca*' function from the kuenm R package [23]. This function extracts the values of each environmental variable for each cell in the study region, calculates the principal components, and re-creates the spatial structure of the raster file. The loadings of our calibration area were used to transform the variables of our transfer region so that both calibration and transfer regions have the same PC axes [24]. We retained the PCs that accounted for >90% of the variance per set for further analyses; specifically, the first three PCs for temperature (i.e., 98.4%), the first two PCs for humidity (i.e., 99.8%), and the first three PCs for soils (93.9%), were kept which gave us a total of eight environmental variables for model calibration (S1 Table).

## Model calibration

Maxent 3.4.1 was used as our working algorithm with which to fit ecological niche models [25, 26]. Maxent is one of the most frequently used programs in distributional ecology, including for ecological niche modeling of infectious diseases [4, 19, 27-31]. Despite misapplications—which have been described elsewhere [32, 33]—Maxent allows users the exploration of multiple parameters to develop different models of niches [23, 34]. Current methodologies assure extensive exploration of features in frameworks that are fully documented and replicable [23, 35, 36].

The calibration function from the kuenm R package was used to apply the Maxent algorithm and create numerous candidate models based on different feature classes, regularization multipliers, and environmental variables [23, 37]. In Maxent, environmental predictors are transformed to feature classes to evaluate their influence in model development (i.e., model gain [26, 38]). Of the five feature classes available (L = linear, Q = quadratic, P = product, T = threshold, H = hinge), we explored all seven combinations of L, Q, and P, because these features are the most similar to physiological response curves (i.e., Gaussian-like responses [39, 40]) and are less problematic for model transfers [41, 42]. The regularization multiplier parameter allows Maxent to be either more permissive or more restrictive in the way that the models fit to the occurrence points in environmental space, a strong determinant of the amount of area identified as suitable [37]. We examined 24 regularization multiplier values: from 0.1 to 2 at increments of 0.1, as well as 5, 8, 10, and 15.

Finally, recent studies have found that, together with feature classes and regularization parameters, environmental variables can be important drivers of output model variance [43, 44] and, therefore, environmental variable selection is worthy of exploration [27, 30]. Although in theory Maxent is able to accord zero weight to uninformative environmental variables, our experience is that removal of those variables changes model characteristics [45]. For the present study, we divided our eight environmental variables with the following criteria: each PC1 for temperature, humidity, and soils should be evaluated with the rest of the variables; PC2 of temperature must always be with its corresponding PC1, and so forth with the other categories (i.e., humidity and soils); PC3 of any of the environmental sets should always be accompanied by PC1 and PC2 of its corresponding set. Using this scheme, we ended with a total of 18 combinations of environmental PCs to be evaluated.

Models were evaluated internally using a random partition of the occurrences, with half used for model calibration and half used for model evaluation [23, 36]. For model evaluation, we used a hierarchical, three-step approach, as recently applied and recommended in other studies [4, 23, 27, 30, 44]. Using the raw model output, we selected those candidate models making predictions that were statistically significant better than random expectations using partialROC [46], followed by models meeting a performance criterion of 5% omission [46, 47]. Finally, the complexity and goodness of fit was assessed using all the occurrences and the logistic output via the Akaike Information Criterion corrected by sample size (AICc), selecting the model with the lowest value [48, 49]. Notice that AICc was used here only as the final selection criterion to choose the simplest models from among the candidate models selected with other metrics [50].

## ENM final models

Parameters of the models selected as described above were used for development of final model outputs using the corresponding functions in the kuenm package, using all available occurrences [43]. Final settings included a logistic output and 10 bootstrap replicates to account for and permit assessment of model variability [4, 51]. We used the median of these bootstrap replicates to represent the final suitable area. Uncertainty from the bootstrapping with the selected parametrization was represented as the interquartile range (IQR) of the replicates on a cell-by-cell basis [52, 53]. Because this uncertainty layer accounts for variability deriving from different occurrences in the bootstrapping process, areas of low uncertainty represent regions of model consistency where predictions are stable to inclusion of different samples of occurrence points.

## ENM model transfers

Models calibrated across areas close to known occurrences were transferred worldwide (except Antarctica); more detailed analyses were focused on the continental U.S. Transfers were

performed using the “no extrapolation” setting in Maxent, which is a conservative approach to interpreting model transfers.

The presence of non-analogous environmental conditions in the transfer area was addressed using the mobility-oriented parity test (MOP; [41]) with the ‘*kuenm_mmop*’ function [23] using 10% reference points from the calibration area for testing [41]. The output of the analysis allows identification of areas of strict extrapolation and different gradients of similarity between the projection and calibration region.

# Results

## Ecological niche modeling (ENM)

Final models chosen from among the 3,024 candidate models took the form of two ‘best’ sets of Maxent parameterizations (S2 Table), with only one set of environmental variables, including PC1 and PC2 from temperature, PC1 from humidity, and PC1 from soil variables. Due to the non-extrapolative nature of the selected model transference method, areas outside environmental calibration ranges are automatically set to zero suitability in all maps presented in the remainder of our analyses. A representation of similarity between the calibration and transfer region can be found in S2 Fig.

Highly suitable regions across the U.S. with low levels of uncertainty were concentrated in the southeastern states. A gap in suitability between the southern and northern areas of Florida is explained by the presence of environments unrepresented in the model calibration area: that is, areas of strict extrapolation. Texas showed the entire spectrum of suitability for *B. pseudomallei* according to our models with low levels of uncertainty, especially in the central and southeastern regions (S3 Fig).

A threshold of 0.6 was used as a cutoff to apply to the continuous model; areas meeting this threshold that fell near recent melioidosis human detections in Texas were chosen for the environmental sampling efforts conducted in November 2020 (Fig 1 in main text).

# References

1. Limmathurotsakul D, Golding N, Dance DA, Messina JP, Pigott DM, Moyes CL, et al. Predicted global distribution of Burkholderia pseudomallei and burden of melioidosis. Nat Microbiol. 2016;1(1). Epub 2016/02/16. doi: 10.1038/nmicrobiol.2015.8. PubMed PMID: 26877885; PubMed Central PMCID: PMCPMC4746747.

2. Peterson AT. Mapping Disease Transmission Risk Enriching Models Using Biogeography and Ecology Preface. Mapping Disease Transmission Risk: Enriching Models Using Biogeography and Ecology. 2014:Ix-+. PubMed PMID: WOS:000362645000001.

3. Boria RA, Olson LE, Goodman SM, Anderson RP. Spatial filtering to reduce sampling bias can improve the performance of ecological niche models. Ecol Model. 2014;275:73-7. doi: 10.1016/j.ecolmodel.2013.12.012. PubMed PMID: WOS:000331508700007.

4. Romero-Alvarez D, Peterson AT, Salzer JS, Pittiglio C, Shadomy S, Traxler R, et al. Potential distributions of Bacillus anthracis and Bacillus cereus biovar anthracis causing anthrax in Africa. Plos Neglect Trop D. 2020;14(3). doi: ARTN e0008131

10.1371/journal.pntd.0008131. PubMed PMID: WOS:000528655400018.

5. Veloz SD. Spatially autocorrelated sampling falsely inflates measures of accuracy for presence-only niche models. J Biogeogr. 2009;36(12):2290-9. doi: 10.1111/j.1365-2699.2009.02174.x. PubMed PMID: WOS:000271902200010.

6. Aiello-Lammens ME, Boria RA, Radosavljevic A, Vilela B, Anderson RP. spThin: an R package for spatial thinning of species occurrence records for use in ecological niche models.

Ecography. 2015;38(5):541-5. doi: 10.1111/ecog.01132. PubMed PMID: WOS:000354179900012.

7. Soberón J, Peterson AT. Interpretation of models of fundamental ecological niches and species' distributional areas. 2005.

8. Barve N, Barve V, Jimenez-Valverde A, Lira-Noriega A, Maher SP, Peterson AT, et al. The crucial role of the accessible area in ecological niche modeling and species distribution modeling. Ecol Model. 2011;222(11):1810-9. doi: 10.1016/j.ecolmodel.2011.02.011. PubMed PMID: WOS:000290839400003.

9. Saupe EE, Barve V, Myers CE, Soberon J, Barve N, Hensz CM, et al. Variation in niche and distribution model performance: The need for a priori assessment of key causal factors. Ecol Model. 2012;237:11-22. doi: 10.1016/j.ecolmodel.2012.04.001. PubMed PMID: WOS:000305863000002.

10. Escobar LE, Romero-Alvarez D, Leon R, Lepe-Lopez MA, Craft ME, Borbor-Cordova MJ, et al. Declining Prevalence of Disease Vectors Under Climate Change. Sci Rep-Uk. 2016;6. doi: ARTN 39150

10.1038/srep39150. PubMed PMID: WOS:000389888300001.

11. Poo-Munoz DA, Escobar LE, Peterson AT, Astorga F, Organ JF, Medina-Vogel G. Galictis cuja (Mammalia): An update of current knowledge and geographic distribution. Iheringia Ser Zool. 2014;104(3):341-6. doi: 10.1590/1678-476620141043341346. PubMed PMID: WOS:000345241300010.

12. Romero-Alvarez D, Escobar LE, Varela S, Larkin DJ, Phelps NBD. Forecasting distributions of an aquatic invasive species (Nitellopsis obtusa) under future climate scenarios. Plos One. 2017;12(7). doi: ARTN e0180930

10.1371/journal.pone.0180930. PubMed PMID: WOS:000405649700061.

13. Dinerstein E, Olson D, Joshi A, Vynne C, Burgess ND, Wikramanayake E, et al. An Ecoregion-Based Approach to Protecting Half the Terrestrial Realm. Bioscience. 2017;67(6):534-45. doi: 10.1093/biosci/bix014. PubMed PMID: WOS:000402826200008.

14. Yañez-Arenas C, Díaz-Gamboa L, Patrón-Rivero C, López-Reyes K, Chiappa-Carrara X. Estimating geographic patterns of ophidism risk in Ecuador. Neotropical Biodiversity. 2018;4(1):55-61. doi: 10.1080/23766808.2018.1454762.

15. Chen PS, Chen YS, Lin HH, Liu PJ, Ni WF, Hsueh PT, et al. Airborne Transmission of Melioidosis to Humans from Environmental Aerosols Contaminated with B. pseudomallei. Plos Neglect Trop D. 2015;9(6). doi: ARTN e0003834

10.1371/journal.pntd.0003834. PubMed PMID: WOS:000357398100036.

16. Hall CM, Jaramillo S, Jimenez R, Stone NE, Centner H, Busch JD, et al. Burkholderia pseudomallei, the causative agent of melioidosis, is rare but ecologically established and widely dispersed in the environment in Puerto Rico. PLoS Negl Trop Dis. 2019;13(9):e0007727. Epub 2019/09/06. doi: 10.1371/journal.pntd.0007727. PubMed PMID: 31487287; PubMed Central PMCID: PMCPMC6748447.

17. Hsueh PT, Huang WT, Hsueh HK, Chen YL, Chen YS. Transmission Modes of Melioidosis in Taiwan. Trop Med Infect Dis. 2018;3(1). doi: 10.3390/tropicalmed3010026. PubMed PMID: WOS:000549485300025.

18. Vega GC, Pertierra LR, Olalla-Tarraga MA. Data Descriptor: MERRAclim, a high-resolution global dataset of remotely sensed bioclimatic variables for ecological modelling. Sci Data. 2017;4. doi: ARTN 170078

10.1038/sdata.2017.78. PubMed PMID: WOS:000403699400001.

19. Campbell LP, Luther C, Moo-Llanes D, Ramsey JM, Danis-Lozano R, Peterson AT. Climate change influences on global distributions of dengue and chikungunya virus vectors. Philos T R Soc B. 2015;370(1665). doi: ARTN 20140135
10.1098/rstb.2014.0135. PubMed PMID: WOS:000350829800012.

20. Escobar LE, Lira-Noriega A, Medina-Vogel G, Peterson AT. Potential for spread of the white-nose fungus (Pseudogymnoascus destructans) in the Americas: use of Maxent and NicheA to assure strict model transference. Geospatial Health. 2014;9(1):221-9. doi: DOI 10.4081/gh.2014.19. PubMed PMID: WOS:000346512600019.

21. Hengl T, de Jesus JM, Heuvelink GBM, Gonzalez MR, Kilibarda M, Blagotic A, et al. SoilGrids250m: Global gridded soil information based on machine learning. Plos One. 2017;12(2). doi: ARTN e0169748
10.1371/journal.pone.0169748. PubMed PMID: WOS:000394424500005.

22. Demsar U, Harris P, Brunsdon C, Fotheringham AS, McLoone S. Principal Component Analysis on Spatial Data: An Overview. Ann Assoc Am Geogr. 2013;103(1):106-28. doi: 10.1080/00045608.2012.689236. PubMed PMID: WOS:000311690700007.

23. Cobos ME, Peterson AT, Barve N, Osorio-Olvera L. kuenm: an R package for detailed development of ecological niche models using Maxent. Peerj. 2019;7. doi: ARTN e6281
10.7717/peerj.6281. PubMed PMID: WOS:000458005300004.

24. Peterson AT, Campbell LP, Moo-Llanes DA, Travi B, Gonzalez C, Ferro MC, et al. Influences of climate change on the potential distribution of Lutzomyia longipalpis sensu lato (Psychodidae: Phlebotominae). Int J Parasitol. 2017;47(10-11):667-74. doi: 10.1016/j.ijpara.2017.04.007. PubMed PMID: WOS:000411773700010.

25. Phillips SJ, Anderson RP, Dudik M, Schapire RE, Blair ME. Opening the black box: an open-source release of Maxent. Ecography. 2017;40(7):887-93. doi: 10.1111/ecog.03049. PubMed PMID: WOS:000405455800010.

26. Phillips SJ, Dudik M. Modeling of species distributions with Maxent: new extensions and a comprehensive evaluation. Ecography. 2008;31(2):161-75. doi: 10.1111/j.0906-7590.2008.5203.x. PubMed PMID: WOS:000254499200001.

27. Alkishe A, Cobos ME, Peterson AT, Samy AM. Recognizing sources of uncertainty in disease vector ecological niche models: An example with the tick Rhipicephalus sanguineus sensu lato. Perspect Ecol Conser. 2020;18(2):91-102. doi: 10.1016/j.pecon.2020.03.002. PubMed PMID: WOS:000552231800034.

28. de Oliveira SV, Romero-Alvarez D, Martins TF, dos Santos JP, Labruna MB, Gazeta GS, et al. Amblyomma ticks and future climate: Range contraction due to climate warming. Acta Trop. 2017;176:340-8. doi: 10.1016/j.actatropica.2017.07.033. PubMed PMID: WOS:000413606800049.

29. Escobar LE, Peterson AT, Papes M, Favi M, Yung V, Restif O, et al. Ecological approaches in veterinary epidemiology: mapping the risk of bat-borne rabies using vegetation indices and night-time light satellite imagery. Vet Res. 2015;46. doi: ARTN 92

10.1186/s13567-015-0235-7. PubMed PMID: WOS:000360528100003.

30. Raghavan RK, Barker SC, Cobos ME, Barker D, Teo EJM, Foley DH, et al. Potential Spatial Distribution of the Newly Introduced Long-horned Tick, Haemaphysalis longicornis in North America. Sci Rep-Uk. 2019;9. doi: ARTN 498

10.1038/s41598-018-37205-2. PubMed PMID: WOS:000456553400064.

31. Samy AM, Annajar BB, Dokhan MR, Boussaa S, Peterson AT. Coarse-resolution Ecology of Etiological Agent, Vector, and Reservoirs of Zoonotic Cutaneous Leishmaniasis in Libya. Plos Neglect Trop D. 2016;10(2). doi: ARTN e0004381

10.1371/journal.pntd.0004381. PubMed PMID: WOS:000372567300014.

32. Escobar LE. Ecological niche models in public health: five crucial questions. Rev Panam Salud Publ. 2016;40(2):98-. PubMed PMID: WOS:000393211200006.

33. Escobar LE, Peterson AT. Spatial epidemiology of bat-borne rabies in Colombia. Rev Panam Salud Publ. 2013;34(2):135-6. PubMed PMID: WOS:000327251100009.

34. Zhu GP, Fan JY, Peterson AT. Schistosoma japonicum transmission risk maps at present and under climate change in mainland China. Plos Neglect Trop D. 2017;11(10). doi: ARTN e0006021

10.1371/journal.pntd.0006021. PubMed PMID: WOS:000414271400068.

35. Kass JM, Vilela B, Aiello-Lammens ME, Muscarella R, Merow C, Anderson RP. WALLACE: A flexible platform for reproducible modeling of species niches and distributions built for community expansion. Methods Ecol Evol. 2018;9(4):1151-6. doi: 10.1111/2041-210x.12945. PubMed PMID: WOS:000429421800032.

36. Muscarella R, Galante PJ, Soley-Guardia M, Boria RA, Kass JM, Uriarte M, et al. ENMeval: An R package for conducting spatially independent evaluations and estimating optimal model complexity for MAXENT ecological niche models. Methods Ecol Evol. 2014;5(11):1198-205. doi: 10.1111/2041-210x.12261. PubMed PMID: WOS:000345762000008.

37. Radosavljevic A, Anderson RP. Making better MAXENT models of species distributions: complexity, overfitting and evaluation. J Biogeogr. 2014;41(4):629-43. doi: 10.1111/jbi.12227. PubMed PMID: WOS:000332781100001.

38. Merow C, Smith MJ, Silander JA. A practical guide to MaxEnt for modeling species' distributions: what it does, and why inputs and settings matter. Ecography. 2013;36(10):1058-69. doi: 10.1111/j.1600-0587.2013.07872.x. PubMed PMID: WOS:000325114500002.

39. Cunze S, Tackenberg O. Decomposition of the maximum entropy niche function - A step beyond modelling species distribution. Environ Modell Softw. 2015;72:250-60. doi: 10.1016/j.envsoft.2015.05.004. PubMed PMID: WOS:000361906400021.

40. Varela S, Anderson RP, Garcia-Valdes R, Fernandez-Gonzalez F. Environmental filters reduce the effects of sampling bias and improve predictions of ecological niche models. Ecography. 2014;37(11):1084-91. doi: 10.1111/j.1600-0587.2013.00441.x. PubMed PMID: WOS:000344645100008.

41. Owens HL, Campbell LP, Dornak LL, Saupe EE, Barve N, Soberon J, et al. Constraints on interpretation of ecological niche models by limited environmental ranges on calibration areas. Ecol Model. 2013;263:10-8. doi: 10.1016/j.ecolmodel.2013.04.011. PubMed PMID: WOS:000322857600002.

42. Qiao H, Feng X, Escobar LE, Peterson AT, Soberon J, Zhu GP, et al. An evaluation of transferability of ecological niche models. Ecography. 2019;42(3):521-34. doi: 10.1111/ecog.03986. PubMed PMID: WOS:000460078900012.

43. Cobos ME, Peterson AT, Osorio-Olvera L, Jimenez-Garcia D. An exhaustive analysis of heuristic methods for variable selection in ecological niche modeling and species distribution modeling. Ecol Inform. 2019;53. doi: ARTN 100983

10.1016/j.ecoinf.2019.100983. PubMed PMID: WOS:000484875200016.

44. Peterson AT, Cobos ME, Jimenez-Garcia D. Major challenges for correlational ecological niche model projections to future climate conditions. Ann Ny Acad Sci. 2018;1429(1):66-77. doi: 10.1111/nyas.13873. PubMed PMID: WOS:000446008300005.

45. Peterson AT, Papes M, Eaton M. Transferability and model evaluation in ecological niche modeling: a comparison of GARP and Maxent. Ecography. 2007;30(4):550-60. doi: 10.1111/j.2007.0906-7590.05102.x. PubMed PMID: WOS:000248965200010.

46. Peterson AT, Papes M, Soberon J. Rethinking receiver operating characteristic analysis applications in ecological niche modeling. Ecol Model. 2008;213(1):63-72. doi: 10.1016/j.ecolmodel.2007.11.008. PubMed PMID: WOS:000255453500005.

47. Anderson RP, Lew D, Peterson AT. Evaluating predictive models of species' distributions: criteria for selecting optimal models. Ecol Model. 2003;162(3):211-32. doi: Pii S0304-3800(02)00349-6

Doi 10.1016/S0304-3800(02)00349-6. PubMed PMID: WOS:000182470200003.

48. Aho K, Derryberry D, Peterson T. Model selection for ecologists: the worldviews of AIC and BIC. Ecology. 2014;95(3):631-6. doi: 10.1890/13-1452.1. PubMed PMID: WOS:000332823100009.

49. Warren DL, Seifert SN. Ecological niche modeling in Maxent: the importance of model complexity and the performance of model selection criteria. Ecol Appl. 2011;21(2):335-42. doi: Doi 10.1890/10-1171.1. PubMed PMID: WOS:000289893500004.

50. Velasco JA, Gonzalez-Salazar C. Akaike information criterion should not be a "test" of geographical prediction accuracy in ecological niche modelling. Ecol Inform. 2019;51:25-32. doi: 10.1016/j.ecoinf.2019.02.005. PubMed PMID: WOS:000467661300003.

51. Peterson AT, Samy AM. Geographic potential of disease caused by Ebola and Marburg viruses in Africa. Acta Trop. 2016;162:114-24. doi: 10.1016/j.actatropica.2016.06.012. PubMed PMID: WOS:000381840600015.

52. Araujo MB, Whittaker RJ, Ladle RJ, Erhard M. Reducing uncertainty in projections of extinction risk from climate change. Global Ecol Biogeogr. 2005;14(6):529-38. doi: 10.1111/j.1466-822x.2005.00182.x. PubMed PMID: WOS:000232772600004.

53. Cobos ME, Osorio-Olvera L, Peterson AT. Assessment and representation of variability in ecological niche model predictions. bioRxiv. 2019.
