# Supplementary figures and images for: Low risk of acquiring melioidosis from the environment in the continental United States
Source: PLoS One. 2022 Jul 29;17(7):e0270997. doi: 10.1371/journal.pone.0270997 (PMC9337633; doi:10.1371/journal.pone.0270997)

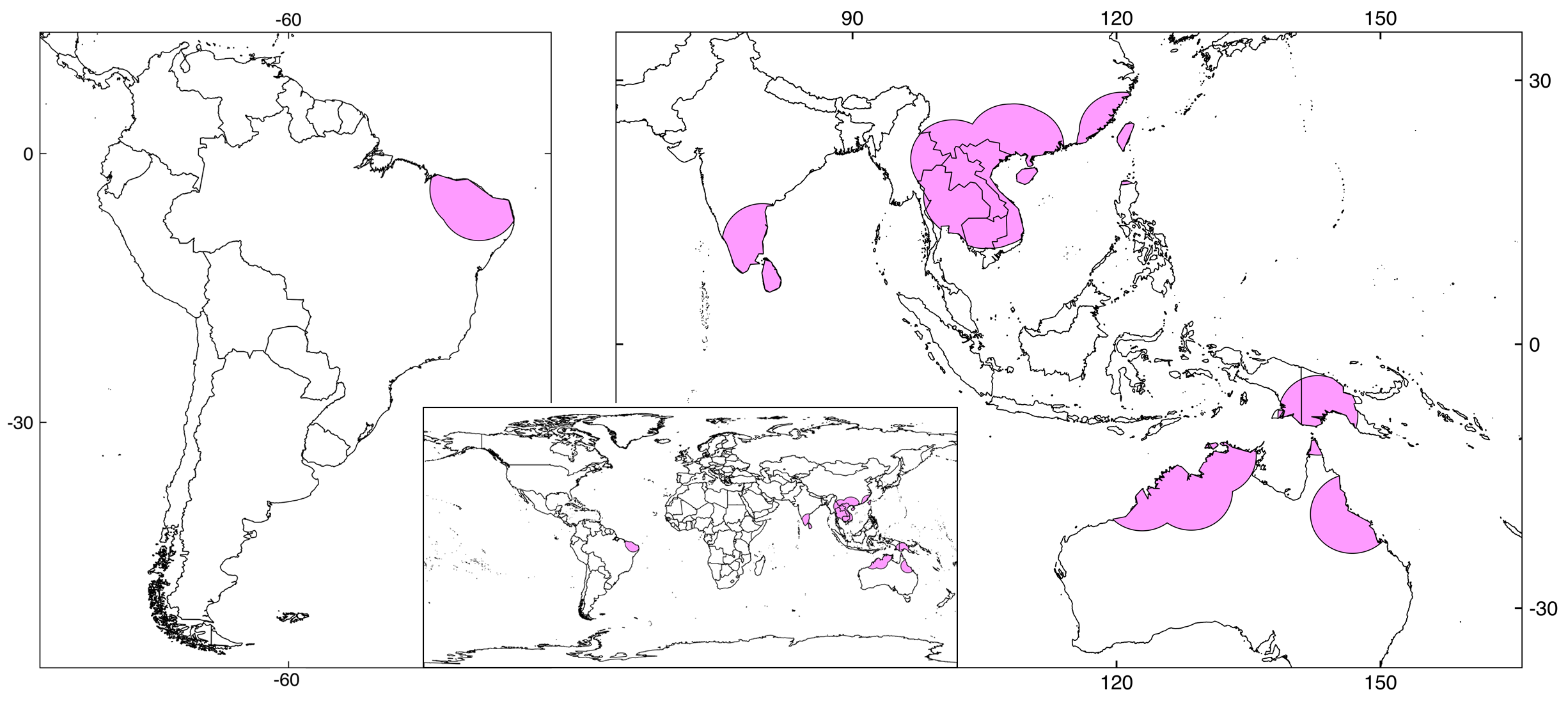

Supplement: S1 Fig — To construct the accessible area used as a model calibration area (pink), we used a ratio of 500 km around 28 recent environmental soil isolations starting in the 2000s obtained from [9]. Location data are available in that reference and can be obtained following the filtering process described in the methods section. (TIF) [file pone.0270997.s002.tif]

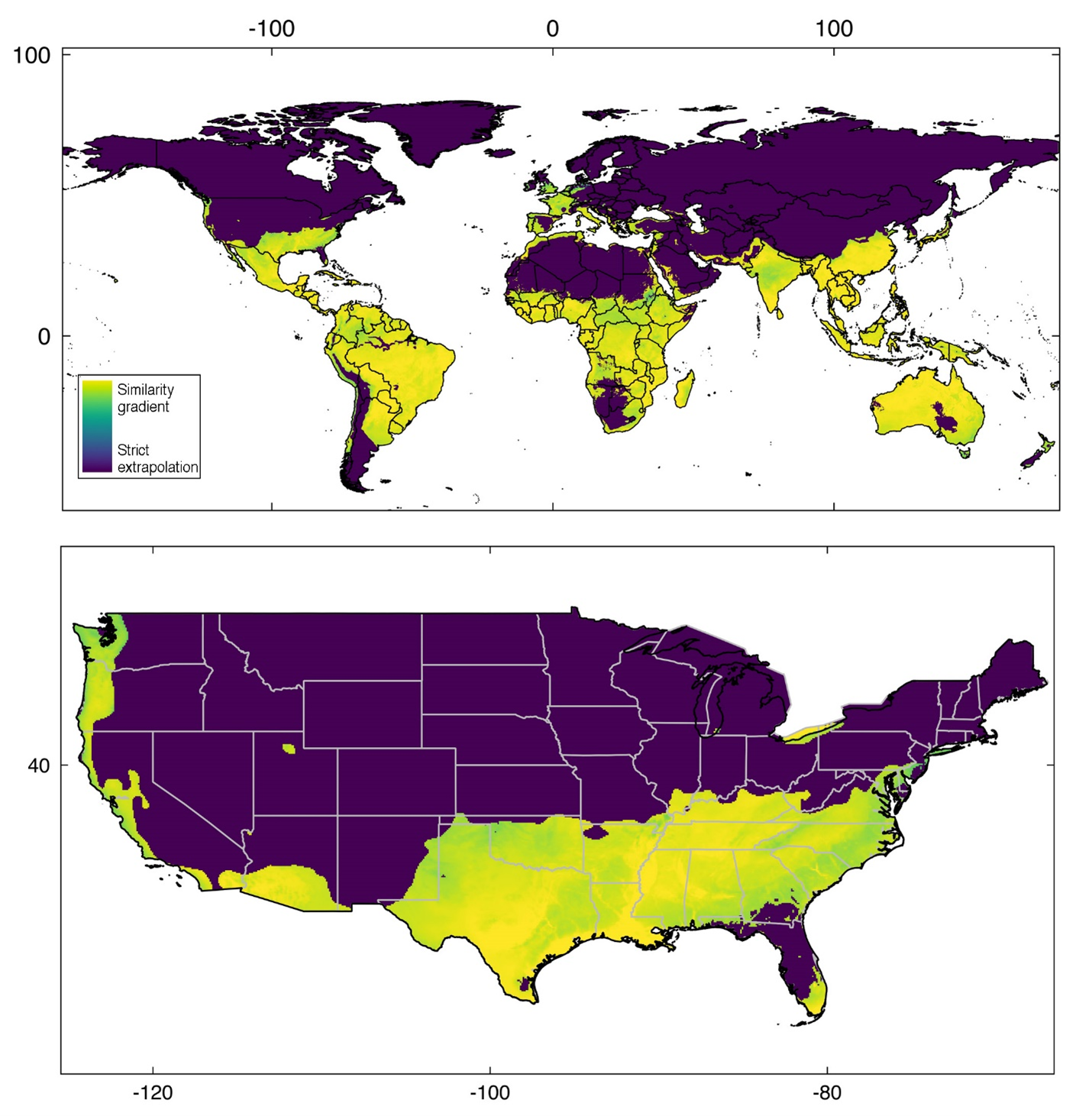

Supplement: S2 Fig — Model interpretation in the purple areas would be highly inadvisable. (TIF) [file pone.0270997.s003.tif]

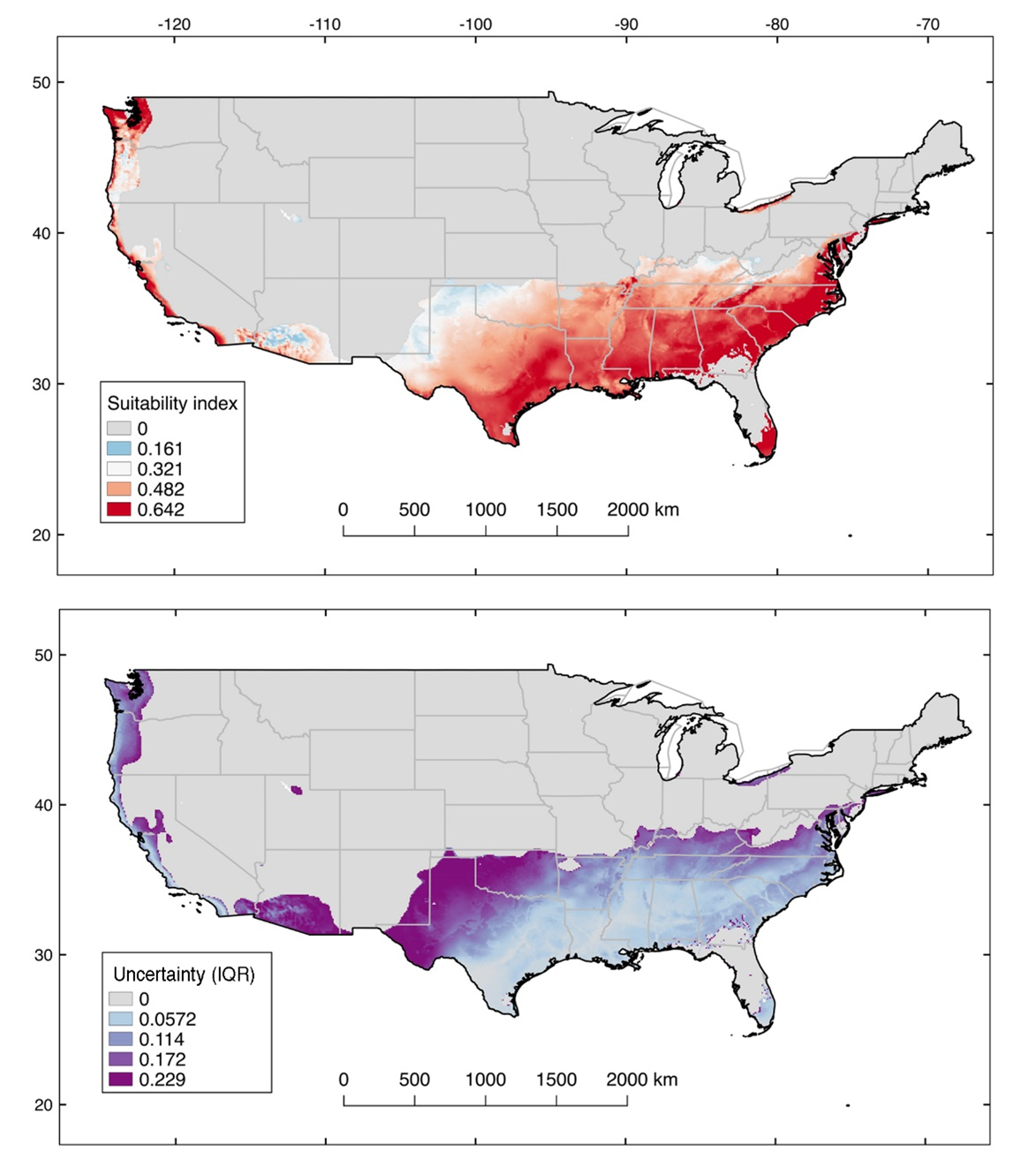

Supplement: S3 Fig — Maps show the continuous model output (upper panel) and the uncertainty is represented as the interquartile range (IQR) among bootstrap replicates (bottom panel). Gray represents areas of strict extrapolation automatically set to zero by the algorithm. (TIF) [file pone.0270997.s004.tif]
