# Supplementary material for: Low risk of acquiring melioidosis from the environment in the continental United States
Source: PLoS One. 2022 Jul 29;17(7):e0270997. doi: 10.1371/journal.pone.0270997 (PMC9337633; doi:10.1371/journal.pone.0270997)
Supplement: S1 Table — PCs included in the analyses are highlighted in gray. (PDF) [file pone.0270997.s005.pdf]

S1 Table. Environmental variables and percent of variance explained by each of the first three principal components (PC1-3). PCs included in the analyses are highlighted in gray. References listed in the table are in S1 File.

Variable set	Description	Source repository (ref.)	PC1	PC2	PC3
Temperature (n = 9)	Bio 1-7, Bio 10-11	MERRAclim (18)	54.8%	33%	10.6%
Humidity (n = 6)	Bio 12-17	MERRAclim (18)	73.8%	25.9%	0.1%
Soils (Depths: 0-5 cm, 15-30 cm, n = 8)	Clay content (Clyppt) Coarse fragments (Crfvol) pH water (Phihox) Sand (Sndppt)	SoilGrids (21)	51.7%	27.1%	15.2%
