# Supplementary material for: Low risk of acquiring melioidosis from the environment in the continental United States
Source: PLoS One. 2022 Jul 29;17(7):e0270997. doi: 10.1371/journal.pone.0270997 (PMC9337633; doi:10.1371/journal.pone.0270997)
Supplement: S2 Table — OR = omission rates, pROC = partial receiving operating characteristic curve (ROC), AICc = Akaike Information Criterion corrected for sample size. (PDF) [file pone.0270997.s006.pdf]

**S2 Table. Maxent parameters for the selected best models and the values corresponding to their evaluation metrics.** OR = omission rates, pROC = partial receiving operating characteristic curve (ROC), AICc = Akaike Information Criterion corrected for sample size.

| Model | Feature type | Regularization parameter | P-value from pROC | OR 5% | AICc | Delta AICc | Number of parameters |
|---|---|---|---|---|---|---|---|
| Model 1 | Product | 1.9 | 0 | 0.0714 | 609.8530 | 0 | 3 |
| Model 2 | Product | 2 | 0 | 0.0714 | 610.063 | 0.2101 | 3 |
